# The Impact of Outgroup Choice and Missing Data on Major Seed Plant Phylogenetics Using Genome-Wide EST Data

**DOI:** 10.1371/journal.pone.0005764

**Published:** 2009-06-02

**Authors:** Jose Eduardo de la Torre-Bárcena, Sergios-Orestis Kolokotronis, Ernest K. Lee, Dennis Wm. Stevenson, Eric D. Brenner, Manpreet S. Katari, Gloria M. Coruzzi, Rob DeSalle

**Affiliations:** 1 Center for Genomics and Systems Biology, Department of Biology, New York University, New York, New York, United States of America; 2 Cullman Molecular Systematics Laboratory and Genomics Laboratory, The New York Botanical Garden, Bronx, New York, United States of America; 3 Sackler Institute for Comparative Genomics, American Museum of Natural History, New York, New York, United States of America; Texas A&M University, United States of America

## Abstract

**Background:**

Genome level analyses have enhanced our view of phylogenetics in many areas of the tree of life. With the production of whole genome DNA sequences of hundreds of organisms and large-scale EST databases a large number of candidate genes for inclusion into phylogenetic analysis have become available. In this work, we exploit the burgeoning genomic data being generated for plant genomes to address one of the more important plant phylogenetic questions concerning the hierarchical relationships of the several major seed plant lineages (angiosperms, Cycadales, Gingkoales, Gnetales, and Coniferales), which continues to be a work in progress, despite numerous studies using single, few or several genes and morphology datasets. Although most recent studies support the notion that gymnosperms and angiosperms are monophyletic and sister groups, they differ on the topological arrangements within each major group.

**Methodology:**

We exploited the EST database to construct a supermatrix of DNA sequences (over 1,200 concatenated orthologous gene partitions for 17 taxa) to examine non-flowering seed plant relationships. This analysis employed programs that offer rapid and robust orthology determination of novel, short sequences from plant ESTs based on reference seed plant genomes. Our phylogenetic analysis retrieved an unbiased (with respect to gene choice), well-resolved and highly supported phylogenetic hypothesis that was robust to various outgroup combinations.

**Conclusions:**

We evaluated character support and the relative contribution of numerous variables (e.g. gene number, missing data, partitioning schemes, taxon sampling and outgroup choice) on tree topology, stability and support metrics. Our results indicate that while missing characters and order of addition of genes to an analysis do not influence branch support, inadequate taxon sampling and limited choice of outgroup(s) can lead to spurious inference of phylogeny when dealing with phylogenomic scale data sets. As expected, support and resolution increases significantly as more informative characters are added, until reaching a threshold, beyond which support metrics stabilize, and the effect of adding conflicting characters is minimized.

## Introduction

Genome level analyses have enhanced our view of phylogenetics in many areas of the tree of life. With the production of whole genome DNA sequences of hundreds of organisms and large-scale EST databases as well as the incorporation of other genome-enhanced technologies [1]–[4], a large number of candidate genes for inclusion into phylogenetic analysis have become available. In this work, we exploit the burgeoning EST database and the steadily growing number of whole plant genomes to address one of the more important phylogenetic questions concerning the hierarchical relationships of the major seed plant lineages (angiosperms, Cycadales, Gingkoales, Gnetales, and Coniferales).

**Figure 1:**
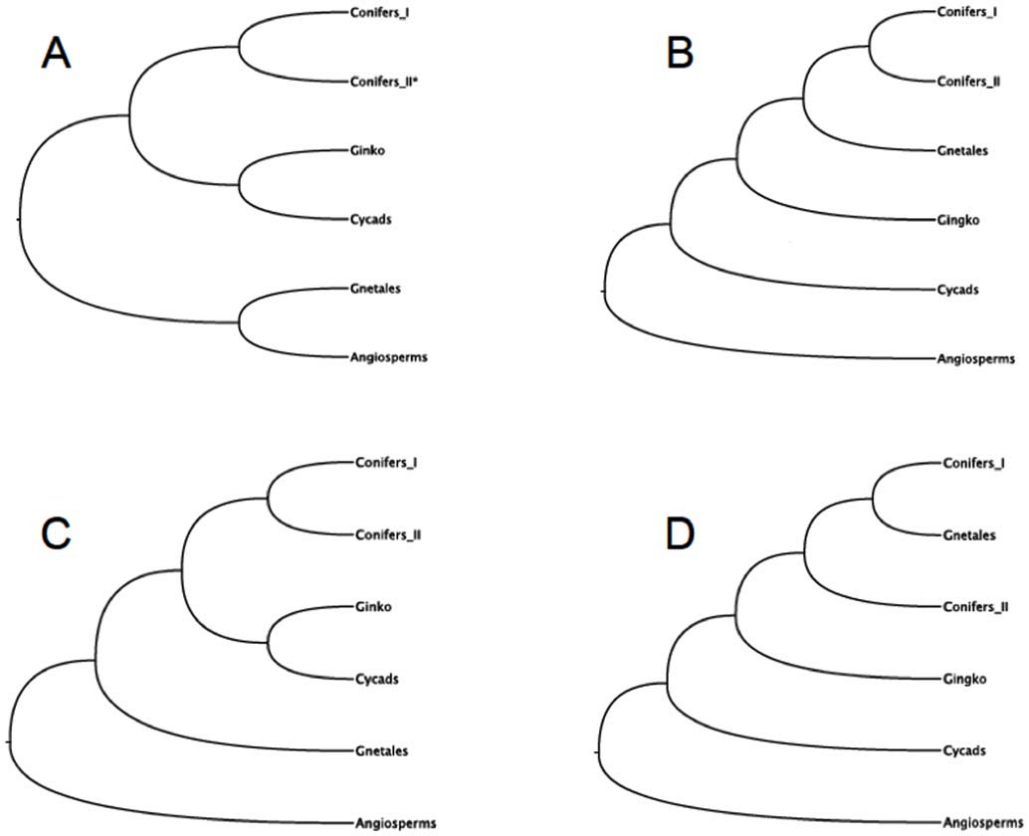
Conflicting phylogenetic hypotheses on the evolution of seed plants. Morphological evidence (synapomophic characterstics shared between angiosperms and Gnetales) have shaped the anthophyte theory, where these two taxa form sister groups (Panel A). In contrast, most molecular studies postulate gymnosperms as a monophyletic group sister to all angiosperms, and place the Gnetales as a sister group to the conifers (Panels B and D). Adding to the controversy, a recent study involving phytochrome genes (Panel C) has placed the Gnetales as basal gymnosperms, with *Ginkgo* and cycads as sister taxa branching after the Coniferales. A: refs. [5], [6], [66]; B and D: refs. [10], [12], [20], [58], [67]; C: ref. [13].

The elucidation of spermatophyte phylogeny continues to be a work in progress, despite numerous studies using single, few or several genes and morphology datasets (morphological: [5]–[9]; and molecular: [10]–[16]) as recently and extensively reviewed [17]. Although most recent studies support the notion that gymnosperms and angiosperms are monophyletic and sister groups, they differ on the topological arrangements within each major group (Figure 1). Many current studies support the placement of Gnetales and conifers as closely-related groups, either as sister clades (Panel B), or with Gnetales as a nested group within the conifers (Panel D). In both of these hypotheses, cycads are the basal clade, followed by *Ginkgo*. A fourth hypotheses, which first emerged through the analysis of the plastid genes *rbc*L and *rpoC1* [18], [19] and multiple plastome genes [20] and again with phytochrome genes [13], [21] and some genes involved in development [16], [22], [23] has generally remained marginal and controversial, places the Gnetales as basal gymnosperms, with conifers and *Ginkgo* plus cycads as later-branching sister groups.

In a previous publication [11], we incorporated Expressed Sequence Tags (ESTs) together with complete protein sequences plus a morphology matrix into a phylogenetic analysis of the seed plants. The concatenation and simultaneous analysis of 43 data partitions yielded a well resolved, single most parsimonious tree with reasonable bootstrap support. In that study we demonstrated the pertinence of using ESTs as a source of phylogenetic characters, provided there is adequate orthology determination. We also stressed the importance of assessing character support in more robust and consistent ways before declaring a phylogenetic question confidently resolved. Given the diverse origins, roles and evolutionary histories of all genes within a particular genome, issues of character support and conflict are relevant when considering the overall history of a taxonomic group, and it appears sensible to consider as many sources of evidence as possible (and available). In this context, the question of where to stop adding characters to a phylogenomic analysis [24] remains open and a high priority for the careful and efficient planning of sequencing projects across all phyla.

Although our earlier approach [11] proved to be very effective in estimating character support and conflict, as well as supporting the case for the use of ESTs in phylogenetic analysis, it was clear more character information was needed to provide stronger support in the resolution of spermatophyte phylogeny. An increase in total characters, but especially an increase in phylogenetically informative characters, would augment both apparent and hidden support in all gymnosperm clades, and provide stronger support for inferences on the hierarchical relationships among the taxa involved. The burgeoning EST and sequencing projects being conducted across genomes make such character information available at an accelerated and sustained pace. One of the main criticisms to phylogenetic projects employing whole- or partial-genome sequences is that with the scarcity of comprehensive genomic or subgenomic data for a large number of taxa, the analyses would retrieve phylogenies for very few taxa that, even if well-resolved and strongly-supported, would represent incorrect evolutionary reconstructions (e.g. [25]). Moreover, Gatesy et al. [26] showed that choice of ingroup taxa at the root of the tree and, more importantly, outgroup choice in deep phylogenomic studies is critical. In the current report, we have expanded taxonomic representation to 17 species, compared to the original six-ingroup, single-outgroup taxa study of de la Torre et al. [11] and expand the number of gene partitions to 1200.

## Materials and Methods

### Orthology prediction

In order to generate a comprehensive molecular matrix to address the phylogenetic questions of flowering versus non-flowering seed plants, we searched the TIGR Plant Transcript Assemblies database (http://plantta.jcvi.org) for well-sampled representatives of all major seed plant groups. Our database search for available EST/unigenes (from a total 226,210 EST assemblies and singletons) from well-sampled representative members of major seed and seed-free plant groups retrieved a total of 158,358 genes from complete genomes (*Arabidopsis*, rice, and poplar), and between 16,000 and 22,000 total unigenes (depending on the dataset) from ESTs for all other species included in various versions of the analysis. In all, the following species were surveyed: *Arabidopsis thaliana, Oryza sativa* (common rice), *Amborella trichopoda*, *Vitis vinifera* (common grape vine), *Populus trichocarpa* (California poplar) (angiosperms); *Cycas rumphii* (Malayan fern palm), *Zamia fischeri*, *Ginkgo biloba*, *Gnetum gnemon* (melinjo, bago, peesae), *Welwitschia mirabilis*, *Cryptomeria japonica* (Japanese cedar), *Pinus taeda* (Loblolly pine) (gymnosperms) as ingroup taxa; *Selaginella moellendorffii* (Lycopophyte), *Adiantum capillus-veneris* (Filicalean fern), *Marchantia polymorpha* (liverwort), *Physcomitrella patens* (moss) and *Chlamydomonas reinhardtii* (unicellular green alga) as outgroups. All available assembled EST databases, independent of their source (tissue, developmental stage, or type of experiment) were surveyed. Using these unigenes, the OrthologID software pipeline ([27]; http://nypg.bio.nyu.edu/orthologid) was employed to predict orthologous groups resulting in fully aligned matrices composed of 926–1,600 gene or ortholog partitions. The variance in the number of orthologs depended on the filtering schemes discussed below. These ortholog groups consisted mostly of translated EST sequence data.

### Ortholog filtering

OrthologID identifies all genes that are orthologous amongst the taxon set under examination [27]. Due to the incomplete nature of the EST database, oftentimes the resulting orthologous groups will include only a few taxa. In addition, the available orthologs can be distributed in specific and narrowly defined taxonomic groups. We reasoned that the inclusion of partitions with three or fewer orthologs will add little to the robustness of the present analysis, so we developed a filtering function in our informatics analysis pipeline that removed any ortholog sets that had fewer than four taxa with genes in the ortholog group. In addition, we restricted the distribution of this filtering to include only those ortholog groups with at least three ingroup taxa (specifically at least two gymnosperms and one angiosperm) and one outgroup taxon per partition. We arrived at a comprehensive dataset formed by 12 ingroup species and 4 outgroup species. We found that using all available outgroups resulted in the retrieval of the largest number of *bona fide* orthologous partitions (1,239) with the filtering scheme specifying the minimal presence of three ingroup taxa (two gymnosperms and one angiosperm) and one outgroup per partition. The resulting ortholog groups comprise genes that are randomly distributed throughout the genome as demonstrated by mapping the loci on the chromosome map of *Arabidopsis thaliana* (Figure S1). This somewhat balances for the general bias of EST and transcriptome data, which most often show enrichment for genes implicated in metabolism, energy and general housekeeping, and an underrepresentation for functional categories such as gene regulation. Still, our dataset comprises an array of orthologous genes belonging to diverse functional categories (Figure S2) including transcriptional regulators and signaling genes. The fact that statistical tests (z-scores, Sungear [28]; data not shown) show a lack of overrepresentation of these categories further suggests that our ortholog sample is more balanced (i.e. less biased) than any previously reported for similar studies of EST data.

### Construction of a comprehensive seed plant phylogenomic matrix

Once the ortholog groups were established as detailed above, we used the Perl script ASAP (Automated Simultaneous Analysis Phylogenies; [29]) to organize and construct a matrix. This program automatically constructs a matrix with named partitions into gene name, GO category, and other informatics categories. The concatenated partitioned matrix can be found in Document S1.

### Phylogenomic analyses

The phylogenetic matrix was analyzed using maximum parsimony (MP) and maximum likelihood (ML) optimality criteria. Parsimony analysis was performed in PAUP* 4b10 [30] using equal weights. Node support was evaluated using the nonparametric bootstrap and jackknife methods in PAUP. Pairwise phylogenetic congruence across all partitions was tested using the ILD test (incongruence length difference; [31], [32]) in PAUP. While this measure has been criticized recently [33]–[36], we choose to use this test conservatively in the context of this study. Branch support measures, such as the Bremer index [37], partitioned branch support [38], and hidden branch support [39], were calculated in ASAP in conjunction with PAUP. Maximum likelihood inference was carried out in RA×ML 7.0.4 [40] at the AMNH Computational Sciences facility on an 8-way server with 2.2 GHz AMD Opteron 846 processors and 128 GB RAM using the fine-grained parallel Pthreads (POSIX Threads Library; [41]) and on the CIPRES cluster (http://www.phylo.org) using the MPI (Message Passing Interface; [42], [43]) implementations. The substitution model best fitting the data was selected in ProtTest [44] by contrasting each model inference's log-likelihood score. The JTT model [45] yielded the highest likelihood score and therefore was used in ML inference taking into account empirical amino acid frequencies calculated directly from the data in hand (Document S2). Among-site rate heterogeneity was accounted for using the CAT approximation model [46] with 25 site rate categories. Node support was quantified with 1625 rapid bootstrap pseudo-replicates as implemented in the parallel versions of RA×ML [47]. In order to explore outgroup choice on tree topology, we performed a series of searches, with different combinations of ingroup and outgroup taxa. These manipulations are summarized in Figure S3. We also explored the effect of missing taxa on the overall phylogenetic hypothesis by measuring the amount of branch support (BS) and partitioned hidden branch support (PHBS) for trees generated by serial nested additions of ingroup taxa (3–11). This analysis involved serially adding partitions with up to 3 taxa, then up to 4 taxa, and so on, so that the matrix kept expanding as partitions with more taxa were added.

## Results

### The impact of outgroup choice on seed plant phylogenetics

**Figure 2:**
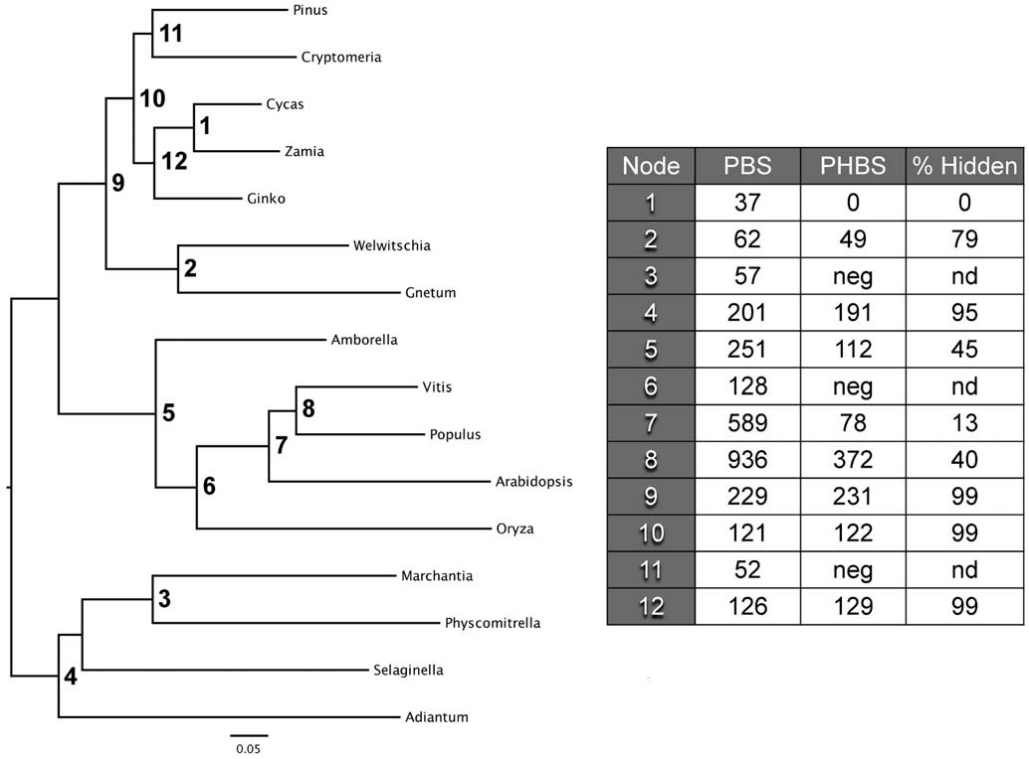
Phylogenetic relationships of seed plants using 1200 genes inferred with parsimony and likelihood methods. The topologies were identical across optimality criteria. The tree shown here was estimated by maximum likelihood using the JTT substitution matrix and empirical amino acid frequencies with the CAT model for among-site rate heterogeneity and final optimization with the GAMMA model. Log-likelihood = −3989109.546056 and *α* (alpha) = 0.720925. The bar denotes 0.05 substitutions/site. All nodes received the highest level of support regardless of the optimality criterion. The table inset shows partition support values (PBS and PHBS). The rightmost column in this table shows the proportion of hidden total support. neg, negative hidden support; nd, non-definable.

In order to address the issue of random rooting [26], [48] we chose to break up the long root to the seed plants by including additional outgroup taxa (*Physcomitrella, Marchantia, Selaginella*, and *Adiantum*). Species chosen to implement this approach fulfilled two criteria: known phylogenetic relevance and good representation in the database. The results are shown in Figure 2. The relative placement of gymnosperm groups changes as outgroup taxa are excluded or rooting is forced on certain seed plant taxa. If no outgroups are specified, trees behave differently depending on whether (and which) seedless taxa are included. When the unicellular green alga *Chlamydomonas* and/or the moss *Physcomitrella* are included, cycads and Ginkgo nest within the conifers, and Gnetales appear basal. When only the heterosporous lycophyte, *Selaginella* (or any of the seed plants) is used to root the tree, Gnetales and conifers group together, and form a sister group to cycads and Ginkgo. Forcing the latter to be the outgroup does not change the relative positions of the former. Gap-coding the matrix results in similar arrangements, except for *Cryptomeria*, which falls outside the gymnosperms – probably due to insufficient amounts of informative characters.

Figure S2 suggests that the effect of long branch attraction or random rooting, can be neutralized by multiple outgroup analysis. In fact, our resulting tree topology remains stable and robust regardless of which outgroup, or outgroup combinations we use (including no outgroup, when rooting with any of the seed plants), suggesting we might have reached a large enough number of informative characters to render a highly robust topology, immune to outgroup choice. In all subsequent analyses we remove *Chlamydomonas* from the analysis due to the fact that it appears to have extreme random root effects [26], [48] and that we have replaced it with four other more appropriate outgroups.

### A robust phylogenomic hypothesis focused on the relationships of major seed plant groups

Phylogenetic analysis of the most inclusive matrix we constructed (72,900 informative characters from 16 species) resulted in a single most parsimonious tree with very high measures of branch support. Figure 2A shows the MP tree of 12 seed plant ingroup taxa rooted with all four outgroup taxa (non-seed plants). Bootstrap and jackknife support values are all at or near 100%. Bremer decay values vary, but all are above double-digits. Higher-level inferences of relationships are consistent with most previous molecular analyses, showing gymnosperms as a monophyletic group sister to the angiosperms. As expected, angiosperm species conform to the well-accepted view that *Amborella* is basal to all flowering plants, followed by the separation between monocots (*Oryza*) and the eudicots *Arabidopsis*, *Vitis*, and *Populus* [25], [49]. Not surprisingly, as two of these species are fully sequenced, all measures of support for angiosperm groupings are very high (Bremer indices in the triple-digits).

The grouping of gymnosperms in the expanded analysis shown in Figure 2A is different from the one observed in our previous study [11], which placed cycads as the earliest diverging branch followed by *Ginkgo*, and then the Gnetales and conifers as sister taxa deeper in the gymnosperm clade (i.e., a pectinate gymnosperm clade). We point out that in the present study, the tree generated differs from the previous one not only in the overall number of taxa, where the ingroup is doubled, and the outgroup is quadrupled, but also in the overall placement of gymnosperm taxa. The MP tree (Figure 2A) shows *Gnetum* and *Welwitschia* (which form a solid monophyletic group) branching early and forming a sister clade to all other gymnosperms.

Notably, the topology of the phylogenomic tree shown in Figure 2A does not agree with two prior hypotheses. The first proposes that all conifers are sister to Gnetales, and the second proposes that the Gnetales are nested within the conifers in particular, placed as sister to conifers I (e.g. [10]; see Figure 1B and 1D). In addition, our initial hypothesis [11] that cycads, followed by *Ginkgo*, could be the earliest diverging extant gymnosperms is not supported in this larger analysis. Instead, the present analysis seems to provide robust support for the hypothesis that Gnetales are the earliest diverging gymnosperm lineage (Figure 1C), previously postulated using phytochrome genes as data sources [13] and in other analyses using the chloroplast gene *rpoC1* [19], using the *AGL*6 [16], [22], and using *Floricaula*/*LEAFY* [23] even though they are the most recent group in the seed plant fossil record. Figure 2 shows the maximum likelihood (ML) tree that agrees entirely with the MP tree topology. This tree has robust (100%) likelihood bootstrap values at all nodes with the exception of the node supporting the clade (*Selaginella*,(*Marchantia*,*Physcomitrella*)) at 54%. The final log-likelihood score and branch lengths were optimized with the GAMMA model of rate heterogeneity in RA×ML and yielded a score of −3989109.546056 and an *α* (alpha) shape parameter of the Γ (Gamma) distribution of 0.720925.

### Missing taxa have a significant effect on tree topology and support – relevance to EST phylogenomics

Previous studies using both simulated (e.g. [50]) and real (using ESTs: e.g. [51], [52]) datasets have tested whether large amounts of missing taxa have a significant effect on the topology and support of a phylogenetic analysis. This type of analysis is particularly relevant to EST studies as the probability of obtaining a full complement of taxa for a particular ortholog is reduced as the number of taxa in the analysis increases (see [53] for an example in animals). This approach is generally accomplished by comparing support metrics and topology changes on datasets with and without given combinations of missing taxa. All existing results (with little change in these factors for compared datasets) have hitherto suggested that large numbers of missing taxa *per se* do not alter either the signal or support values. However, when “missing taxa” also means too few available characters for a correct call regarding taxon placement, the negative effect is indeed dramatic.

Our analysis on the 43-partition matrix [11] revealed that subtracting partitions with high taxon representation did collapse many branches or significantly lower overall support, although the exclusion of these taxon-dense partitions also meant the removal of crucially informative character information. We explored the effect these missing taxa had on the overall phylogenetic hypothesis by comparing the amount of branch support and hidden branch support for each node using partitions where information was available for 7, 6, 5, and 4 taxa.

**Figure 3:**
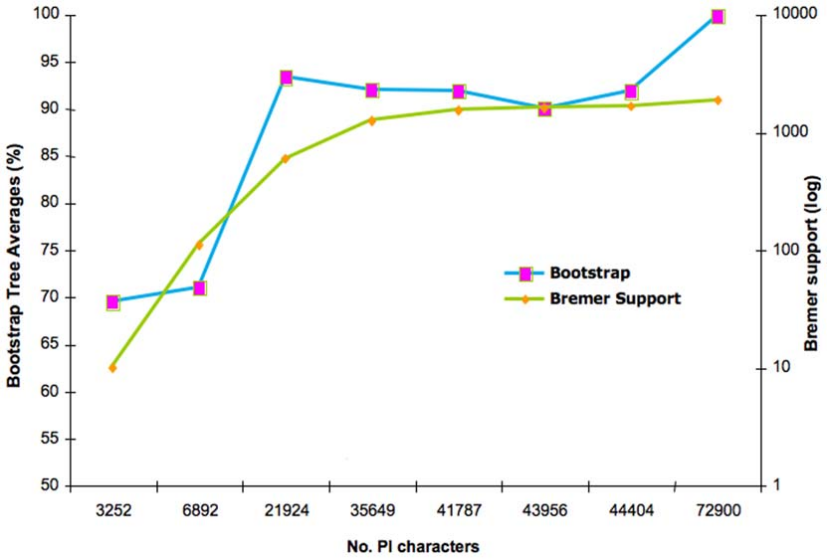
Measures of tree support plateau as character information grows beyond a threshold. Plot of the bootstrap value averaged over all nodes in trees (Y axis on the left) obtained for randomly chosen PI character set sizes (X axis). Included is a plot of total Bremer support value in trees (Y axis on the left) obtained for randomly chosen PI character set sizes (X axis). Bremer support reaches a clear plateau after roughly 36,000 PI characters, while bootstrap values reach a peak at 21,000 PI characters, then oscillate at 90–95%, and reach a 99–100% maximum average around 73,000 PI characters.

As shown in Figure 3, tree support values increase dramatically as more partitions with fuller taxon complements are added. This result could argue for the exclusion of partitions with low number of taxa. When analyzing individual partitions, it is clear that trees from those with lower number of taxa have fewer informative characters, number of resolved clades and, ultimately, lower support value across the board. However, we also suggest keeping those partitions with even minimal character information, as these partitions may often prove valuable in the resolution of a single clade or clades within the tree.

**Figure 4:**
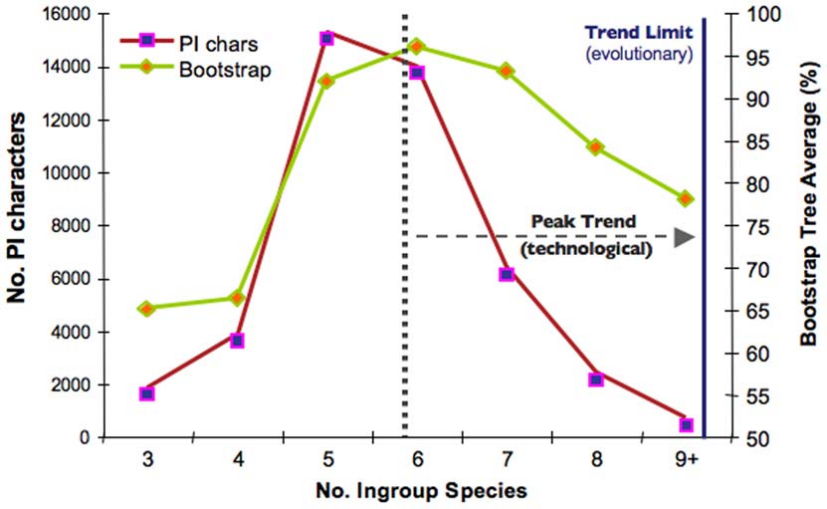
Character information and tree support values reach a threshold at a compromise point between sequence and taxon sampling space. Result of plotting the number of parsimony informative characters and bootstrap values for varying number of taxa. To generate this figure we binned the genes with 3, 4, 5, 6, 7, 8, and 9 ingroup taxa. Next we estimated the number of parsimony informative characters for all of the genes in those bins (plotted on the Y axis on the left) and the bootstrap values for nodes for the trees generated from the genes in each bin (the average for all nodes in the trees is given on the Y axis on the right). Note that the number of PI characters drops drastically as the number of taxa passes 6 for both measures. This trend is the result of fewer and fewer genes having fuller taxonomic representation. That is, there are far fewer genes with 9 ingroup taxa represented than genes with 6 taxa and hence many fewer parsimony informative characters. The dotted line represents what we call a “technological limit” for the present study. As more and more EST sequences and whole genome sequences are added to databases, this limit will move to the right (as indicated by the dotted arrow).

We also explored the effect of missing taxa on the overall phylogenetic hypothesis. Figure 4 depicts how the data in our study relates to the compromise of increasing number of character and taxa. Given our choice of taxa, and the current sequence availability for each species (indicated on the X axis of Figure 4), a peak of informative characters and related bootstrap values (a “phylogenetic sweet spot” of sorts) is reached between 5 and 6 taxa. That is genes that are found in five or six of the taxa in this study when combined have more parsimony informative characters and higher overall bootstrap values This result is attained as a result of there being fewer and fewer genes with fuller taxonomic representation in the EST database.

This result does not necessarily mean the incorporation of additional taxa is of no value. Potentially important character information is still obtained when adding more taxa. While this illustrates the effect of missing taxa for genes in the EST database, an analysis will benefit from the compounded information obtained from including all partitions containing 3 to 9 ingroup taxa, below and above which phylogenetic information will be null. In theory, the upper limit will shift to the right as more genomes are sequenced, until reaching an absolute limit, given by evolutionary – not technological – constraints, i.e. a real lack of overlap for several genes among species. As seen before, even when adding incomplete partitions (i.e. with varying amounts of taxon representation within the partition), support increases radically as more parsimony-informative sequence data are added. This result indeed argues for the inclusion of all information available, as long as a minimum of 3 ingroup and one outgroup species is maintained in each partition.

### Analysis of individual partitions

As shown previously for seed plants [11] and yeast species [24], analysis of trees generated with individual data partitions, reveals large disagreement with the simultaneous analysis tree hypothesis. Yet, as shown in earlier studies (e.g. [11], [54], [55]), most, if not all, of such apparent incongruence is statistically significant using the ILD test. We employed this test in order to explore the interaction among data partitions within our dataset and the degree of incongruence at the character level. Due to computational constraints within PAUP, we limited the number of individual pairwise comparisons, and generated random samples of paired ILD comparisons corresponding to 10% of the total dataset, and performed pairwise ILD tests on this random sample of combinations of these subsets (data not shown).

We evaluated the effect of increasing character information (PI, parsimony-informative) on both bootstrap and Bremer support values. Figure 3 reflects a definite overall increase in bootstrap metrics as the number of PI characters go up, but shows different behaviors for each. This trend continues without a clear limit or plateau. The variance makes sense, as the very nature of these metrics changes as a function of the addition of new data partitions with varying degrees of supporting and conflicting character information. By contrast, traditional Bremer support values, show an overall upward trend but reach a clear plateau after the 900 partition-mark (∼30,000 PI characters), and remain unchanged even after more data partitions are added. This trend holds well above the 40,000 PI character-mark (Figure 3).

Bootstrap support values show a slightly different trend (Figure 3, and Figure S4). Bootstrap averages rise steadily at first and then plateau within a limited range between 91 and 96% past the 780-partition mark (∼20,000 PI characters) even as many more PI characters continue to be added. This result again suggests enough character information is present in the matrix to support the concatenated tree topology in >90% bootstrap replicates, but enough conflicting information is present to account for mild oscillations. Near-100% bootstrap and jackknife values are reached in most tree nodes (e.g. trees in Figure 2). Inclusion of differing character information in a concatenated approach is still preferred as a more accurate approximation to the true species phylogeny, as evidenced by the retrieval of a single, total evidence tree with high support values even though large amounts of significantly conflicting data (data not shown) are present in the combined dataset.

### Partitioned analysis reveals the behavior of character support and conflict

By using partitioned support metrics, both hidden and apparent, we were able to identify those individual partitions contributing various degrees of positive, negative or null support to the all-evidence topology (Figure 2B). Most partitions (>50%) contribute no hidden support to the concatenated analysis tree, while roughly 22% contribute positive hidden support, and about 15% contribute negative support to the simultaneous analysis tree. This means only 1/6 of all data partitions contain characters that actually conflict with the concatenated analysis hypothesis and result in worse tree length scores, although less than half (i.e. ∼8%) of total partitions actually contribute more than three steps of negative hidden support. In contrast, more than half (i.e. >12%) of the partitions contribute more than three steps of positive hidden support to the simultaneous analysis hypothesis.

## Discussion

### Implications for seed plant phylogeny

While our initial approach used for 43 partitions and 7 seed plant species in a previous study [11] may have been appropriate to explore the utility of EST data in phylogenetic analysis, limited taxon sampling and choice of outgroups most definitely influenced the retrieval of a conflicting topology to that presented here which is based on >1,200 partitions from 16 plant species. The fact that we have used a relatively unbiased EST sampling method, the sheer number of informative characters and additional taxa, and the various tests for robustness described earlier, all make us prefer either of the current trees to any previous phylogenetic hypotheses for the seed plants. This result also supports a long-standing observation that high bootstrap values reflect the local concordance of the topology with the data, but provide little indication of the approximation of a particular topology/dataset to the true species phylogeny. Two completely different topologies reflecting relationships among comparable groups of data may both have equally high bootstrap values, and still fall far from the true species tree [24].

While our current hypothesis still reveals a few branches lacking in robustness – a problem that will most likely be solved by adding more sequence data from currently under-represented species – our analysis nonetheless puts forward several well corroborated hypotheses concerning seed plant phylogeny, namely:

- Gymnosperms are a monophyletic group, sister to the angiosperms.
- *Amborella* is confirmed as a basal angiosperm, sister to monocots and eudicots.
- Gnetales and conifers are separate, monophyletic groups, (i.e. not nested within one another).
- The clade formed by cycads and *Ginkgo* share a common ancestor.

Additionally, our results suggest Gnetales may indeed be the sister group to the rest of the extant gymnosperms. While it is conceivable that further taxon addition may falsify this hypothesis in the future, the high support values for the tree in Figure 2, together with our observations in serial addition experiments, supports the basal placement of Gnetales within the gymnosperms. Furthermore, alternative hypotheses using conflicting partitions and partition sets are poorly resolved, do not agree on a particular alternative, and generally receive poor support values.

While the topology of the Gnetales as sister to the rest of the gymnosperms may be considered unconventional, it is quite interesting to note that this topology has been retrieved from individual gene trees such as *rpoC1* and *rbc*L as well as the noncoding regions of the inverted repeat representing the plastome [18]–[20] and the nuclear genome using phytochrome genes [13], [21], and agamous genes *AGL6* and *AGL-like* genes [16], [22] and *Floricaula*/*LEAFY* [23]. Besides representing multiple genes from two different genomes, these data also represent a diversity of functions within the plants. Therefore, it should come as no surprise that this topology could be supported by our data set. Moreover, the analysis represents a substantial data set that is not only consistent with a basal position for Gnetales either as sister to all other seed plants or as sister to the rest of the gymnosperms but also when analyses include bryophyte, lycophyte and pteridoyphyte outgroups as for example in the analysis of *rbc*L data [56]. It should also be noted that the ages of known fossils are minimum ages so the young age for Gnetales is simple that a minimum age.

### Impact of outgroup choice

The observation that a denser ingroup taxon sampling did not have a major effect on tree topology beyond a certain point, but a change in outgroup taxa identity and number did make gymnosperm relationships vary significantly, stimulated us to look at outgroup choice in more detail. This problem is an issue observed in previous studies yet largely overlooked in the literature regarding this group [10]. In general, this issue has not been addressed in a systematic way, either because not all gymnosperm groups have been included, or because not enough taxa have been sampled (e.g. [10], [57], [58]). Alternatively, the problem is avoided altogether by rooting with a seed plant – either an angiosperm or with what are usually considered the more primitive gymnosperms (i.e. Gnetales; see Figure 2). Overall, our data indicate that outgroup choice can severely influence tree topology in datasets with lower numbers of informative characters, but that the addition of more informative characters can lead to a point where outgroup choice plays a minimal role.

### Support and the seed plant phylogenetic tree

Due to the incomplete nature of EST information, both as it refers to the absence of full-length sequences as well as to the randomness of sequencing for each species sampled, many of our partitioned matrices had different degrees of missing data. Throughout the present analysis, we evaluated the effect of missing characters and missing taxa on tree topology and branch support. We conclude that increasing taxon sampling is crucial in retrieving precise and unambiguous phylogenies, and outgroup choice can be a determining factor in resolving controversial phylogenies by minimizing the effect of long branch attraction. In contrast, missing characters do not seem to play a significant role in altering support metrics, as long as informative characters are present to resolve species relationships. Similarly, gene order does not appear to be a determining factor, while the effect of gene identity becomes less and less significant as the number of (randomly-selected) partitions increase. Ultimately, and as a representative sample of the species' genomes is approached, this variable will end up playing a minimal, marginal role in influencing support values.

A single EST-based tree may have well supported clades that have reached a limit – or plateau – of support (such as nodes 4–8) coexisting with poorly supported nodes (e.g. 1, 2, 11), which do not have enough character information to support them. These comparisons also suggest how relative the character-to-support relationship may be. For instance, *Zamia* and *Amborella*, both with ∼14% matrix representation, do not change their position relative to *Cycas* or other angiosperms, respectively, while *Pinus* and *Cryptomeria* (with >80% and ∼15%, respectively) still struggle to “find their place”, *vis-à-vis* each other within the gymnosperms.

### The impact of missing data on approaching a support plateau

Rokas et al. [24] clearly show a plateau of support values for trees as sequence information increases. Among the many imperfections of dealing with an EST-based alignment matrix such as the one in this study, is the randomness of sequencing, which results in suboptimal taxa representation for many of the individual gene partitions. However, this shortcoming allows us to visualize the behavior of various stages of character density that result (as seen earlier) in varying degrees of branch support. By evaluating the effect of taxon and character density produced by the randomness of the EST approach, we can evaluate the degree of support on branches with different character-to-taxon ratios (Figures 3 and 4).

The yeast study [24], and the seed plant hypothesis presented here, both suggest that studies with similar number of taxa may require different numbers of characters and genes in order to reach similar robust topological inferences and high levels of support. This discrepancy is probably a factor of the different phylogenetic scales and divergence times of the groups involved: ingroup taxa in the yeast phylogeny diverged between 50 and 100 million years ago (Mya) [59] and were confined within a single genus. In contrast, the ingroup taxa in our plant study are at the level of families – if not orders – that diverged no earlier than 400 Mya [60]–[62]. Alternatively, tree balance dynamics may have an impact on resolution [63] or as several studies with much larger numbers of ingroup taxa [39], [64], [65] suggest, larger numbers of characters than those of the yeast study are required for robust resolution of some simple phylogenetic hypothesis.

## Supporting Information

Figure S1 Chromosomal location of the 1200 EST orthologs in Arabidopsis used in this study. The Arabidopsis accession number is shown to the left of each linkage group. The figure demonstrates that the ESTs used in this study are dispersed across the entire genome of Arabidopsis.
(0.62 MB TIF)

Figure S2 Histogram showing functional categories for the 528 genes that have GO annotations in the 1200 EST orthologs. Number of ESTs is on the X-axis and GO category is on the Y-axis. This figure demonstrates that many of the ESTs we use in this study are dispersed across very broad GO categories.
(0.75 MB TIF)

Figure S3 Effect of rooting and choice of outgroup in the internal topology of the Spermatophyta. The relative placement of gymnosperm groups changes as outgroup taxa are excluded or rooting is forced on certain seed plant taxa. If no outgroups are specified, trees behave different depending on whether (and which) seedless taxa are included. When the unicellular green alga Chlamydomonas and/or the moss Physcomitrella are included, cycads and Ginkgo nest within the conifers, and Gnetales appear basal. When only the heterosporous fern Selaginella (or any of the seed plants) is used to root the tree, Gnetales and conifers group together, and form a sister group to cycads and Ginkgo. Forcing the latter to be the outgroup does not change the relative positions of the former. Gap-coding the matrix results in similar arrangements, except for Cryptomeria, which falls outside the gymnosperms - probably due to insufficient amounts of informative characters. This figure shows the results of rooting experiments and reveals a crucial role for outgroup choice in tree topology. (A, B) suggest a long-branch attraction effect may be at play. When no outgroup taxa are used, (i.e. rooting with a seed plant; D, E)) or only the fern Selaginella (the closest to seed plants) is specified as outgroup (C), the monophyly of conifers is restored while the bootstrap consensus tree is unresolved for many gymnosperm clades. When outgroup taxa closer to the ingroup are used, Adiantum and Selaginella alone or together do not rescue the monophyly of conifers (G, H).
(0.13 MB TIF)

Figure S4 Effect of taxon addition on branch support metrics. The graph shows the dramatic effect on all support metrics of including more species in the analysis, and indicates our matrix has reached bootstrap support thresholds with the current taxonomic representation. Unlike Bremer support, which plateaus after a threshold of PI characters is reached, partitioned support metrics show an upward trend as new characters with varying support and conflict for the tree are added. As more partitions per taxa (and PI characters) are added, tree resolution improves greatly, from a complete polytomy with partitions including only 4 taxa, to complete resolution starting at 6 taxa per partition, and increasing in branch support values from thereon (data not shown).
(0.10 MB TIF)

Document S1 Nexus file of concatenated partitioned EST matrix.
(1.46 MB ZIP)

Document S2 Empirical amino acid frequencies. Empirical amino acid frequencies calculated directly from the protein alignment.
(0.00 MB TXT)
