# Supplementary figures and images for: The Impact of Outgroup Choice and Missing Data on Major Seed Plant Phylogenetics Using Genome-Wide EST Data
Source: PLoS One. 2009 Jun 2;4(6):e5764. doi: 10.1371/journal.pone.0005764 (PMC2685480; doi:10.1371/journal.pone.0005764)

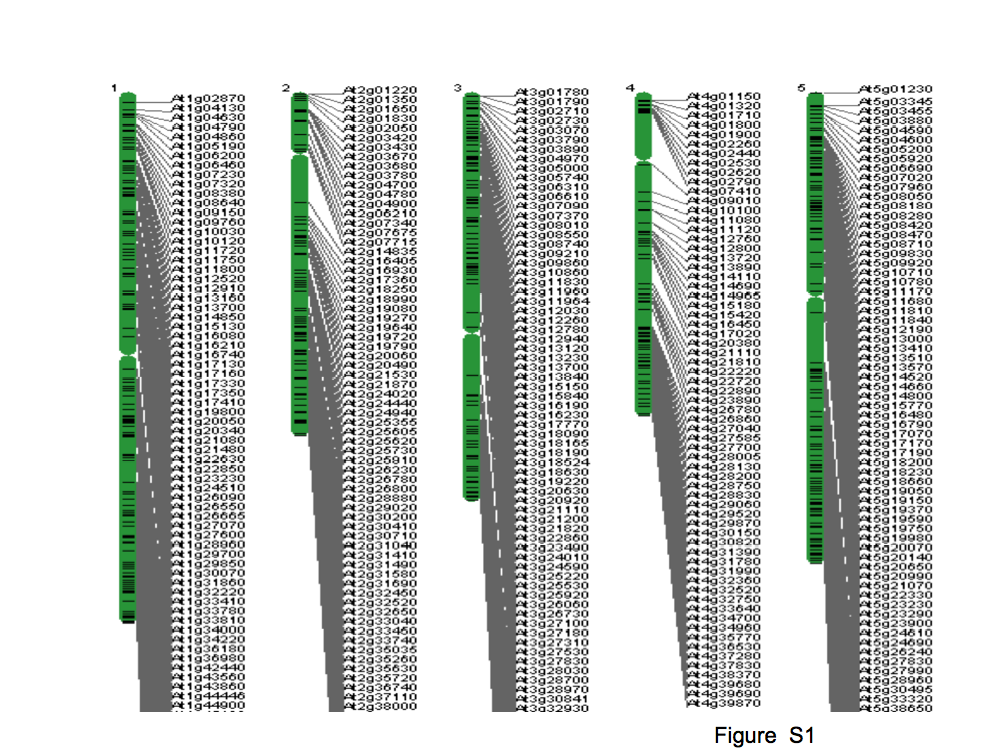

Supplement: Figure S1 — Chromosomal location of the 1200 EST orthologs in Arabidopsis used in this study. The Arabidopsis accession number is shown to the left of each linkage group. The figure demonstrates that the ESTs used in this study are dispersed across the entire genome of Arabidopsis. (0.62 MB TIF) [file pone.0005764.s001.tif]

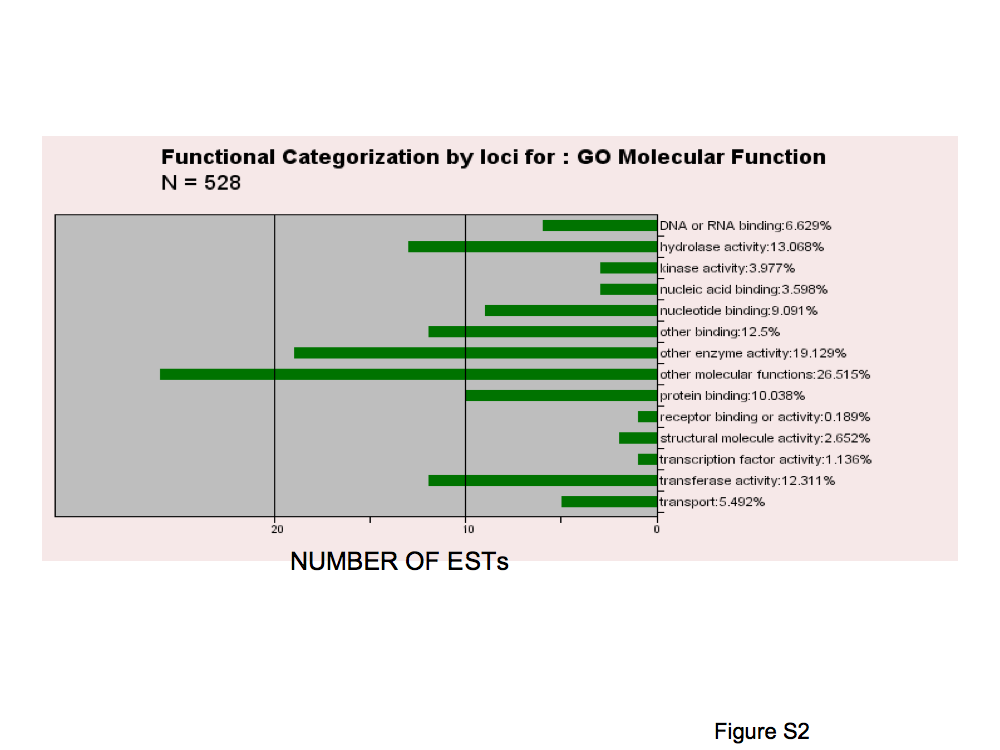

Supplement: Figure S2 — Histogram showing functional categories for the 528 genes that have GO annotations in the 1200 EST orthologs. Number of ESTs is on the X-axis and GO category is on the Y-axis. This figure demonstrates that many of the ESTs we use in this study are dispersed across very broad GO categories. (0.75 MB TIF) [file pone.0005764.s002.tif]

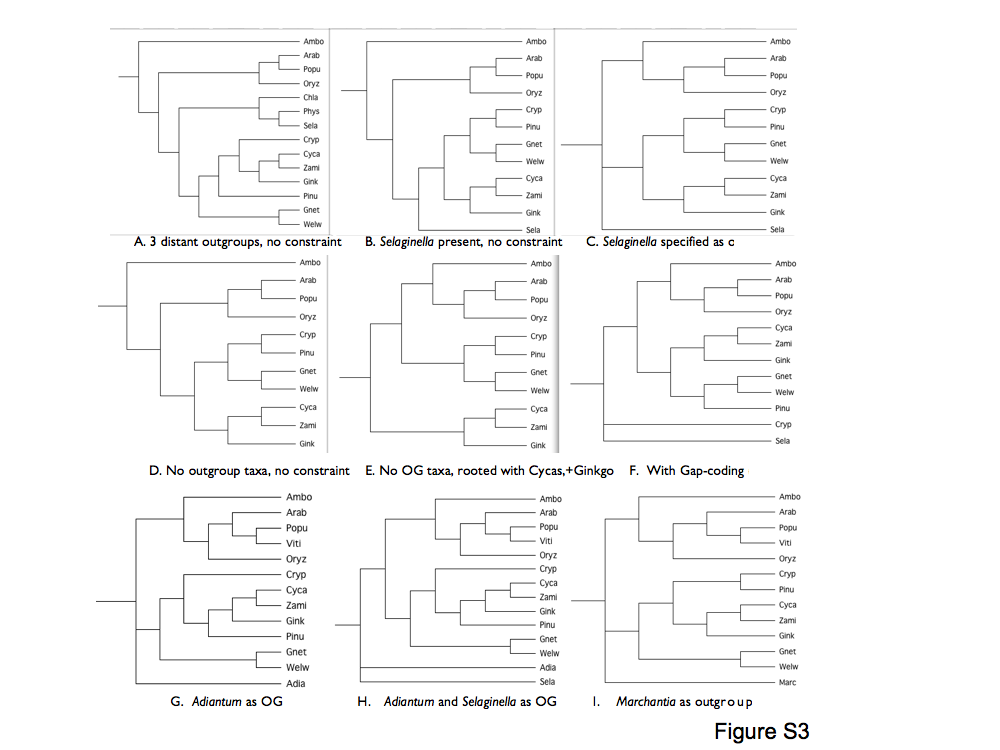

Supplement: Figure S3 — Effect of rooting and choice of outgroup in the internal topology of the Spermatophyta. The relative placement of gymnosperm groups changes as outgroup taxa are excluded or rooting is forced on certain seed plant taxa. If no outgroups are specified, trees behave different depending on whether (and which) seedless taxa are included. When the unicellular green alga Chlamydomonas and/or the moss Physcomitrella are included, cycads and Ginkgo nest within the conifers, and Gnetales appear basal. When only the heterosporous fern Selaginella (or any of the seed plants) is used to root the tree, Gnetales and conifers group together, and form a sister group to cycads and Ginkgo. Forcing the latter to be the outgroup does not change the relative positions of the former. Gap-coding the matrix results in similar arrangements, except for Cryptomeria, which falls outside the gymnosperms - probably due to insufficient amounts of informative characters. This figure shows the results of rooting experiments and reveals a crucial role for outgroup choice in tree topology. (A, B) suggest a long-branch attraction effect may be at play. When no outgroup taxa are used, (i.e. rooting with a seed plant; D, E)) or only the fern Selaginella (the closest to seed plants) is specified as outgroup (C), the monophyly of conifers is restored while the bootstrap consensus tree is unresolved for many gymnosperm clades. When outgroup taxa closer to the ingroup are used, Adiantum and Selaginella alone or together do not rescue the monophyly of conifers (G, H). (0.13 MB TIF) [file pone.0005764.s003.tif]

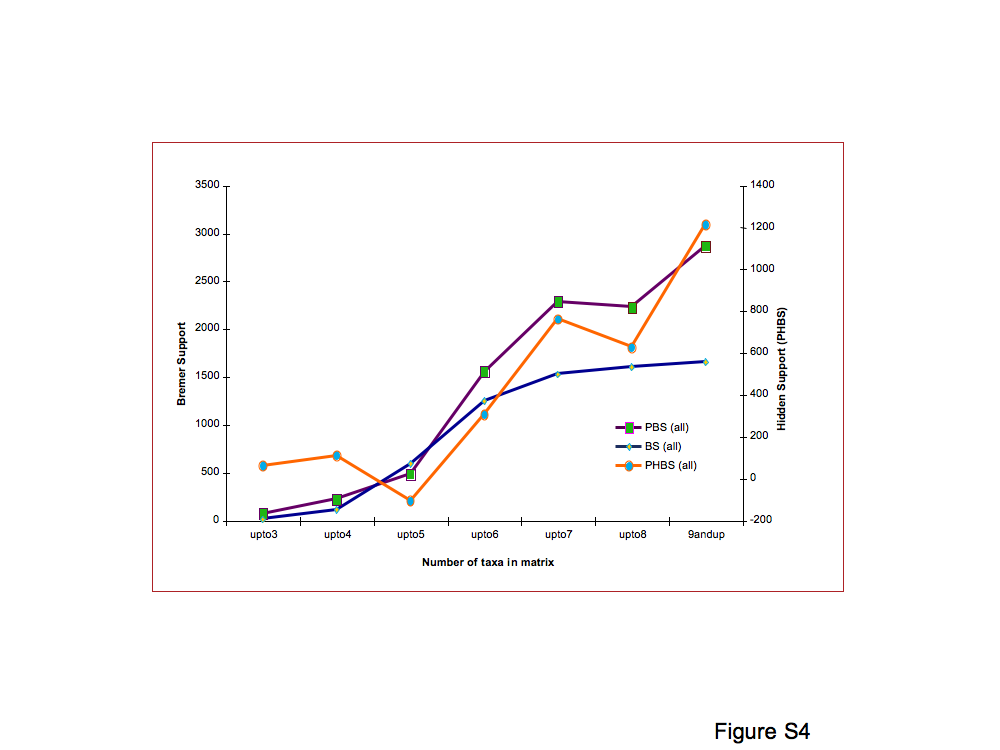

Supplement: Figure S4 — Effect of taxon addition on branch support metrics. The graph shows the dramatic effect on all support metrics of including more species in the analysis, and indicates our matrix has reached bootstrap support thresholds with the current taxonomic representation. Unlike Bremer support, which plateaus after a threshold of PI characters is reached, partitioned support metrics show an upward trend as new characters with varying support and conflict for the tree are added. As more partitions per taxa (and PI characters) are added, tree resolution improves greatly, from a complete polytomy with partitions including only 4 taxa, to complete resolution starting at 6 taxa per partition, and increasing in branch support values from thereon (data not shown). (0.10 MB TIF) [file pone.0005764.s004.tif]
